# An EEG study on artistic and engineering mindsets in students in creative processes

**DOI:** 10.1038/s41598-024-63324-0

**Published:** 2024-06-11

**Authors:** Yuan Yin, Ji Han, Peter R. N. Childs

**Affiliations:** 1 Dyson School of Design Engineering, Imperial College London, London, UK; 2 Department of Innovation, Technology, and Entrepreneurship, University of Exeter, Exeter, UK

**Keywords:** Cognitive control, Consciousness, Decision, Problem solving, Human behaviour

## Abstract

This study aims to take higher-education students as examples to understand and compare artistic and engineering mindsets in creative processes using EEG. Fifteen Master of Fine Arts (MFA) visual arts and fifteen Master of Engineering (MEng) design engineering students were recruited and asked to complete alternative uses tasks wearing an EEG headset. The results revealed that (1) the engineering-mindset students responded to creative ideas faster than artistic-mindset students. (2) Although in creative processes both artistic- and engineering-mindset students showed Theta, Alpha, and Beta wave activity, the active brain areas are slightly different. The active brain areas of artistic-mindset students in creative processes are mainly in the frontal and occipital lobes; while the whole brain (frontal, oriental, temporal, and occipital lobes) was active in creative processes of engineering-mindset students. (3) During the whole creative process, the brain active level of artistic-mindset students was higher than that of engineering-mindset students. The results of this study fills gaps in existing research where only active brain areas and band waves were compared between artistic- and engineering-mindset students in creative processes. For quick thinking in terms of fluency of generating creative ideas, engineering students have an advantage in comparison to those from the visual arts. Also, the study provided more evidence that mindset can affect the active levels of the brain areas. Finally, this study provides educators with more insights on how to stimulate students’ creative ability.

## Introduction

Creativity can regarded as the ability to imagine something novel and valuable^1,2^. Creative mindsets can reflect the perceived source and nature of creativity^3^. Researchers have found there are two kinds of creative mindsets: growth and fixed mindsets^4^. A growth creative mindset, which can support creative skills, is developable with time and practice; while a fixed creative mindset supports that creative skills are fixed and cannot be changed^5^. To maintain a higher creative ability, both creative mindsets are needed^3^.

Researchers have realized the importance of education in developing creative ability^6–9^. Creative abilities can be trained through education, such as elementary, secondary education, and higher education. In elementary education contexts, children are taught what creativity is and apply their creative ability to solve daily problems. However, within the education continuum, creativity of students becomes more domain-specific^9^. Kaufman and Beghetto^10^ support the view that although both artists and engineers are creative, artists perform better in perceiving shapes and colors while engineers perform better in calculations and trigonometry.

Various mindsets have been proposed and characterised. Artistic mindsets are an open-minded and creative mindset to think about and express the world and life through perspectives of aesthetics^11^. Engineering mindsets are a problem-solving mind, where people try to understand how things work and solve problems in a creative but reasonable way^12^. Creative mindsets have been found as an essential element in artistic and engineering mindsets^13^ for it can help people with artistic and engineering mindsets to generate more innovative outcomes.

Researchers have been interested in understanding artistic and engineering mindsets for a long time^7^. By understanding the differences between the two mindsets, the bridges between engineering and the humanities can be understood^14–16^. Although Haller and Courvoisier^17^ claimed that there are no differences between artistic and engineering mindsets^7,16,18^, much existing research still supports the view that there are some differences^19^. Husdon^20^ found that engineering-mindset people may have a stronger ability in convergent thinking while artistic-mindset people may have a stronger ability in divergent thinking^21^. Furnham and Crump^22^ support the view that artistic-mindset students are more open to new things but they have a poor performance on cautiousness compared with engineering-mindset students. In addition, artists are more effective, emotionally unstable, and less socialized while engineers are more conscientious^23^.

Some research has been conducted to investigate the differences between artistic and engineering mindsets in students in creative processes. Van Broekhoven et al.^9^ recruited Arts degree and STEM domain students to perform divergent thinking tasks. In each task, they were asked to generate as many ideas as they could for pointed problems. The results showed the differences in divergent thinking tasks between Arts and STEM students. Arts degree students were more likely to connect novelty with creativity while STEM domain students tend to combine feasibility and effectiveness with creativity. Kaufman et al.^24^ employed students to conduct a five-factor personality measure, a brief self-report of creativity, and the Compound Remote Associates Task (CRAT). The results revealed that engineering-mindset students assess their personal creativity at a lower level compared with artistic-mindset students^10,24^. Hartlet and Greggs^17^ explored students in performing divergent thinking tasks and assessing the outcomes. The results indicated that artistic-mindset students have better performance in the quantity of creative ideas generated than that of engineering-mindset students^25^. Sagone and Caroli^26^ employed students to perform the Thinking Style Inventory and Test of Creative Thinking. They pointed out that art-background students can express their ideas in a more comprehensive way compared with engineering-background students. Feist^23^ asked students to solve problems and conduct personality inventories. The results revealed that artistic-background students have a higher score in creative performance than engineering-background students.

The different performances can be explained from the different focused points between artistic and engineering mindsets. People with artistic mindsets are more likely to create art, such as drawing and painting. They assume that novelty^8^ and aesthetics are important for arts^7^. This leads to the advantages of artistic-mindset students in developing creative mindsets^8^. While engineering-mindset students need to respond to existing requirements, assuming that novelty, effectiveness, and feasibility are all important for solutions^27^. This difference means artistic-mindset students are more likely to link novelty with creativity while engineering-mindset students are more likely to link effectiveness and feasibility with creativity. Researchers also explained the differences in mindsets from Kirton's adaptor-innovator theory^28^. Based on the theory, creativity can consist of innovation and adaptation. Kim^29,30^ has specified that innovation is related to fluency and originality while adaption is related to elaboration and titles. Engineering-mindset students tend to achieve creativity as innovators while artistic-mindset students tend to be adaptors.

Although researchers have tried to explore the differences between artistic and engineering mindsets in creative processes and indicated that the two mindsets are related to cognitive processes, most of the empirical studies were based on asking participants to conduct creative tasks, test their personality, and assess their performance during the creative tasks. Neuroscience technologies (such as electroencephalograph (EEG), Functional magnetic resonance imaging (fMRI), and magnetoencephalogram (MEG), which can directly report human cognitive conditions, have the potential to support understanding of such mindsets. Andreasen and Ramchandran^31^ employed participants with artistic and engineering mindsets to perform word association tasks. During the process, fMRI was used to detect brain conditions. The results indicated that both mindsets participants use the lingual gyrus and cuneus during creative tasks. Some research also focused on understanding a single mindset in creative processes. Ahad et al.^32^ recruited ten mechanical engineering participants to understand the engineering-mindset students’ brain conditions in creative processes. Participants were required to justify whether the given function is a creative function of the target words. EEG was applied to detect the brain conditions, and the N400 effect and decrease of alpha waves were observed during the creative process in the parietooccipital temporal area.

Combinations of artistic and engineering mindsets can help people generate more creative ideas. However, it may be still challenging for people who have either an artistic or engineering mindset to understand the other mindset. This may be because people are not able to identify the differences between two mindsets in cognitive processes of creativity clearly. Considering EEG can be used to understand the cognitive status of people, it has the potential to record different cognitive conditions of artistic and engineering mindsets of people. By comparing the different results, EEG may further help humans understand the differences between artistic and engineering mindsets in creative processes from cognition processes perspectives in depth. After understanding the different cognitive processes, people with artistic or engineering mindset may be able to understand the other mindset more easily. Thus, they may be able to transfer to the other mindset temporarily more easily and cooperate with other mindset people more easily.

These existing studies supported the feasibility of understanding the difference between artistic and engineering mindsets using neuroscience technologies. However, the existing research mainly related to detecting which part of the brain or which band was more active when artistic and engineering mindsets students finish a creative task. More brain activities conditions differences such as event-related potentials, power spectral density, and brain state series have not been fully detected.

**Figure 1:**
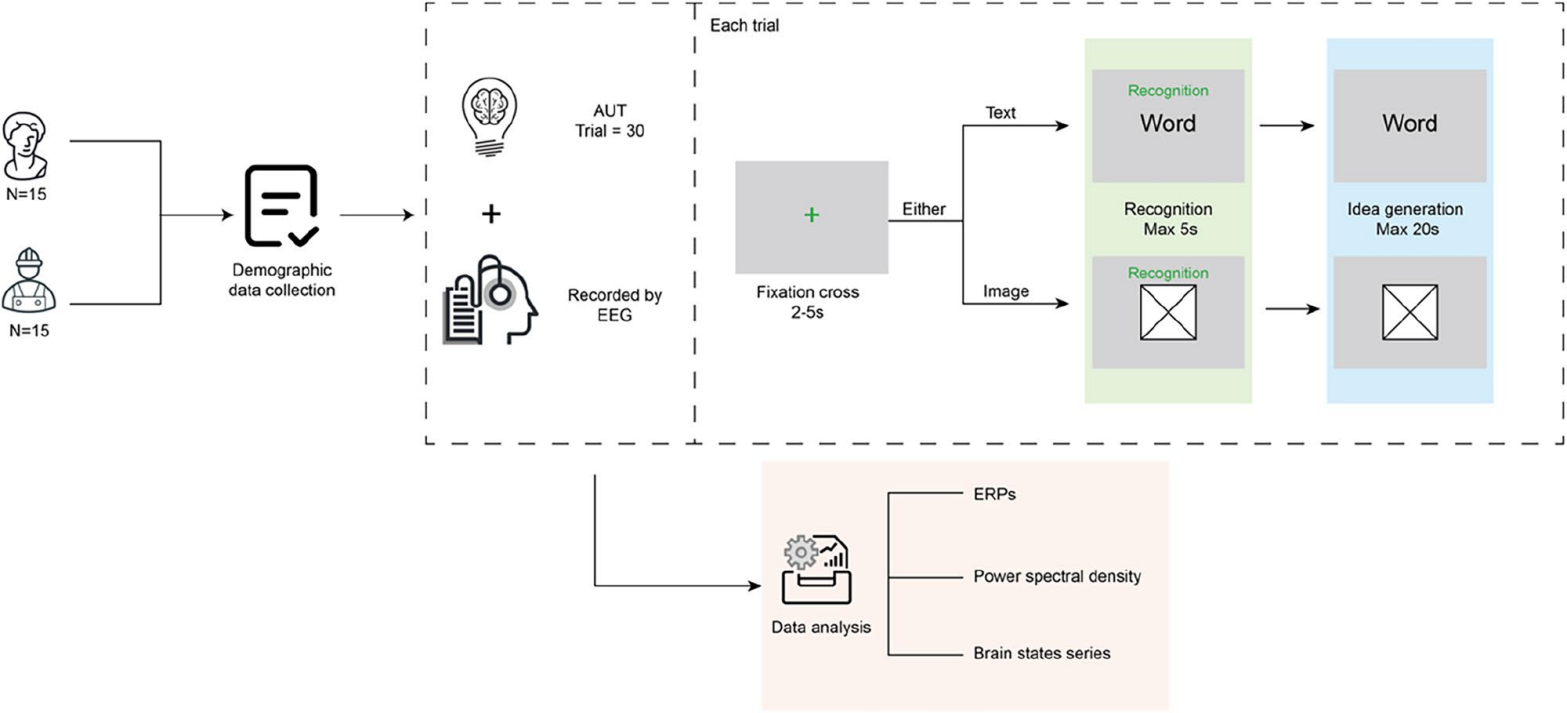
Procedure of the study.

The preceding review of existing research leads to a gap that the cognitive processes between artistic and engineering mindsets in creative processes are not fully explored through EEG methods. Therefore, this study aimed to take higher-education students as examples to understand and compare artistic and engineering mindsets in creative processes using EEG. To be specific, in the study, artistic- and engineering-mindset students were recruited to complete alternative uses tasks (thinking of a use of the given everyday objective that only a few people would think of). During the study, students need to wear a sixteen-channel EEG device to record their brain activities. Channels Fp1/Fp2/F7/F8/F3/F4 report signals on the frontal lobe, C3/C4/P3/P4 report signals on the parietal lobe, T3/T4/T5/T6 report signals on the temporal lobe, and O1/O2 report signals on the occipital lobe. After the study, the event-related potentials (ERP), power spectral density (PSD), and brain states series were analysed to provide useful insight into the brain differences behind creative artistic- and engineering-mindset processes. The procedure of this study is depicted in Fig. 1. The study is approved by the ethics committee in the corresponding author’s institution.

## Results

The MATLAB R2022b (The MathWorks, Inc., Natick, Massachusetts, United States) plugin EEGLAB was used to analyse the EEG signals. With the help of the Automated artifact rejection function in EEGLAB, which is based on the “Clean raw data” EEGLAB plugin, both bad data channels and bad portions of data were marked automatically^33^. EEG signals marked as “artifacts” were removed from the analysis. A 50 Hz notch filter was applied to negate the interference of the electrical mains. Then, the signals were passed through a band-pass filter with a pass-band of 0.1–100 Hz^34,35^. The reference electrodes were placed on the left and right mastoid (M1 and M2). The following three analyses on ERPs results, power spectral density, and brain state series were then conducted. The datasets used and/or analysed during the current study are available from the corresponding author on reasonable request.

### ERPs results

**Figure 2:**
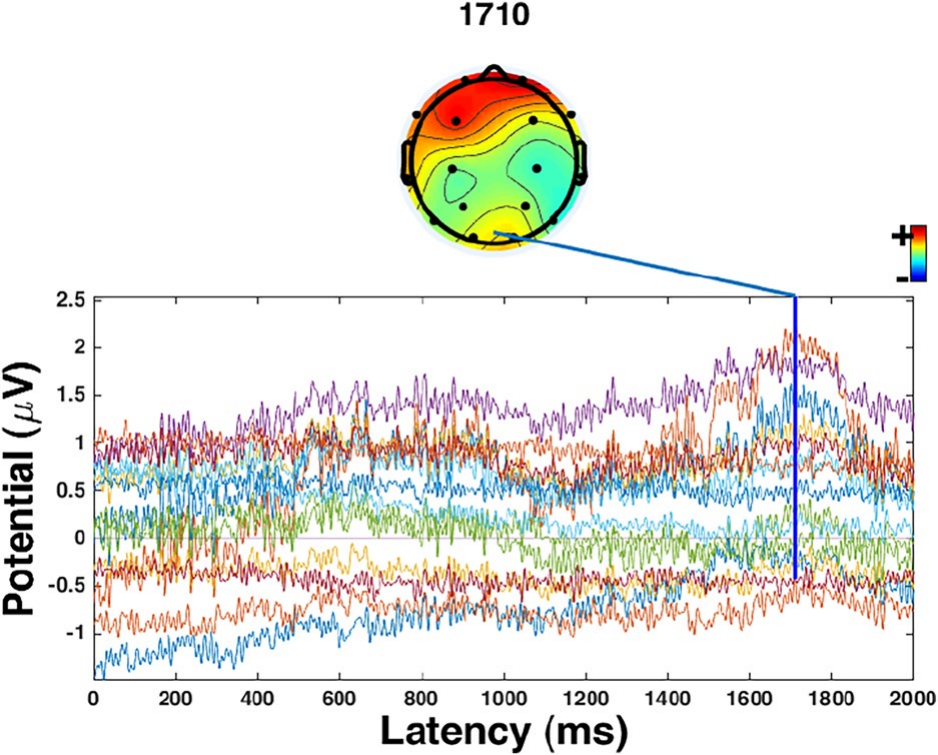
The highest EPR result of MFA visual arts students in creative processes.

**Figure 3:**
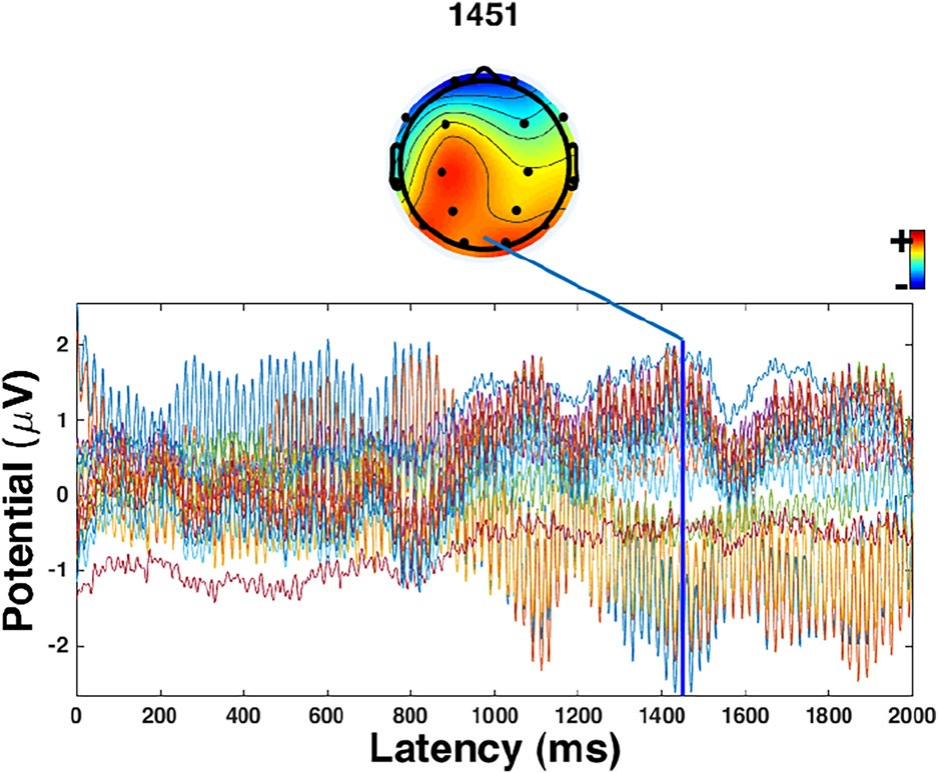
The highest EPR result of MEng design engineering students in creative processes.

ERPs are small voltages generated in the brain, which can quantitatively reflect the brain’s response to a specific cognitive event or stimuli^36,37^. The highest ERP results of MFA and MEng students are shown in Figs. 2 and 3 respectively. Figures 2 and 3 represent the group average brain and the group averages from different channels. The different colors mean the activity levels of the brain. The red color indicates the specific part of the brain has a high measurement active level.The blue color is associated with low or zero activity. From red to blue color, the active level is continuously transited via yellow, green, and cyan color.One-way ANOVA was used to identify whether the ERPs results between MFA visual arts students and MEng design engineering students were significantly different or not. The significant levels set in this study were 0.05.

From the results, the highest EPR result of MFA visual art students is 1710 ms. At this point, Fp1, Fp2, and F3 were most active. Although F7 and O2 were also active, it is not as strong as the former three areas. The highest EPR result of MEng design engineering students is 1451 ms. At this point, C3, P3, T6, O1, and O2 were most active.The highest EPR result of MFA visual arts students (1710 ms) is statistically significantly higher than MEng design engineering students (1451 ms, *p* = 0.042 < 0.05, η_p_^2^ = 0.622).

### Power spectral density

**Figure 4:**
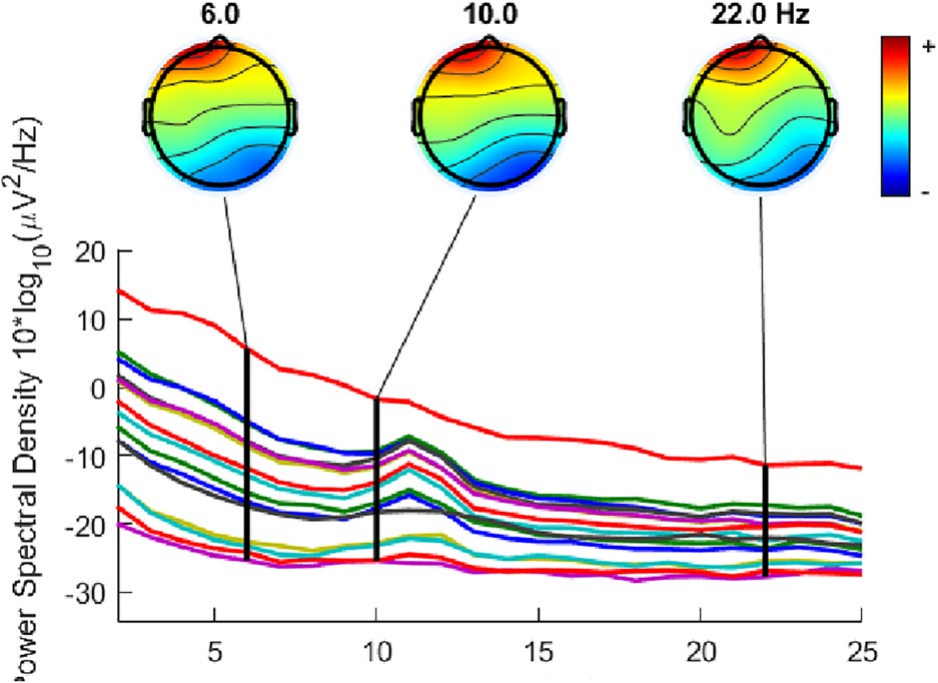
The PSD of MFA visual arts students.

**Figure 5:**
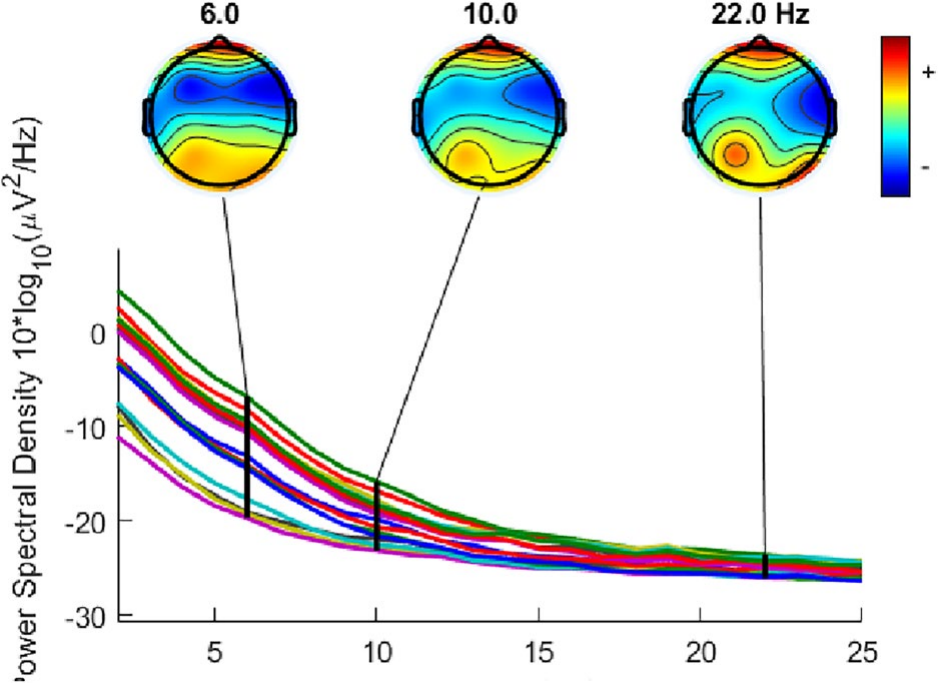
The PSD of MEng design engineering students.

The power spectral density (PSD) is a method to analyse signals’ power content through frequency. Considering the spectra range of theta waves was in 4–8 Hz, Alpha was in 8–12 Hz, and Beta waves was in 12–30 Hz, the middle spectra number of each band wave was selected to represent each band wave^38^. To be specific, 6Hz was selected to represent Theta waves; 10 Hz were selected to represent Alpha waves; 22Hz was selected to represent Beta waves^39^. The PSD results of MFA visual art and MEng design engineering students are displayed in Figs. 4 and 5 respectively. Figures 4 and 5 represent the group average brain and the group averages from different channels. The different colors mean the activity levels of the brain. The red color indicates the specific part of the brain has a high measurement active level. The blue color is associated with low or zero activity. From red to blue colors, the active level is continuously transited via yellow, green, and cyan colors.

From the results, it can be found that for MFA visual arts students, 6 Hz, 10 Hz, and 22 Hz were all strong in Fp1. For MEng design engineering students, although the core active areas in creative processes were Fp1 and Fp2 for all three band wave frequencies, it has some slight differences. 6Hz shows a strong activity in Fp1, Fp2, P3, P4, O1, O2, T5, and T6. 10Hz shows a strong activity in Fp1, Fp2, and P3. 22Hz shows a strong activity in Fp1, Fp2, P3, O2, and T6.

### Brain states series

**Figure 6:**
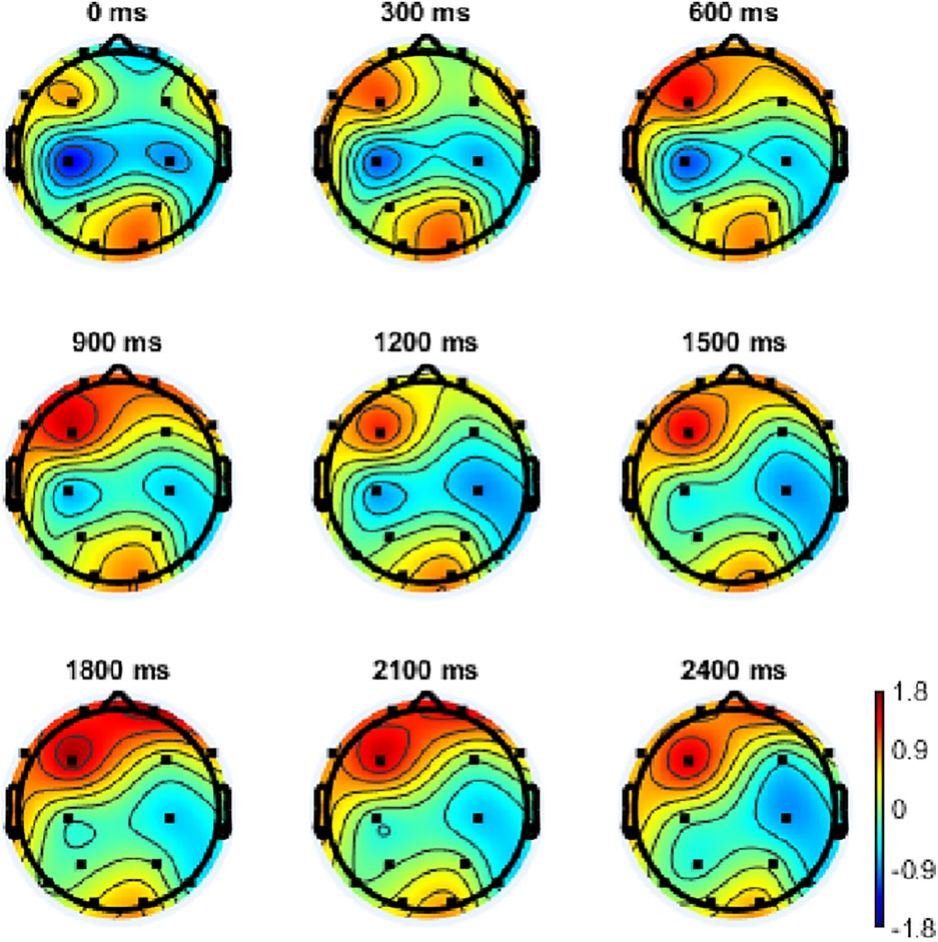
The brain states series of MFA visual arts students.

The brain states dynamic changes were then detected. The results showed the brain states series of each 300 ms in the first 2400 ms. The results of MFA visual arts students (Fig. 6) revealed that in a creative process, the F3, F8, P3, and O2 of the brain were slightly active initially. Then F3 kept active in the whole process and had the strongest active at around 1800 ms. O2 was also active but the activation was reduced slowly and not as strong as F3 during the whole process. Fp1 and Fp2 were active in some periods such as 900 ms and 1800–2100 ms.

**Figure 7:**
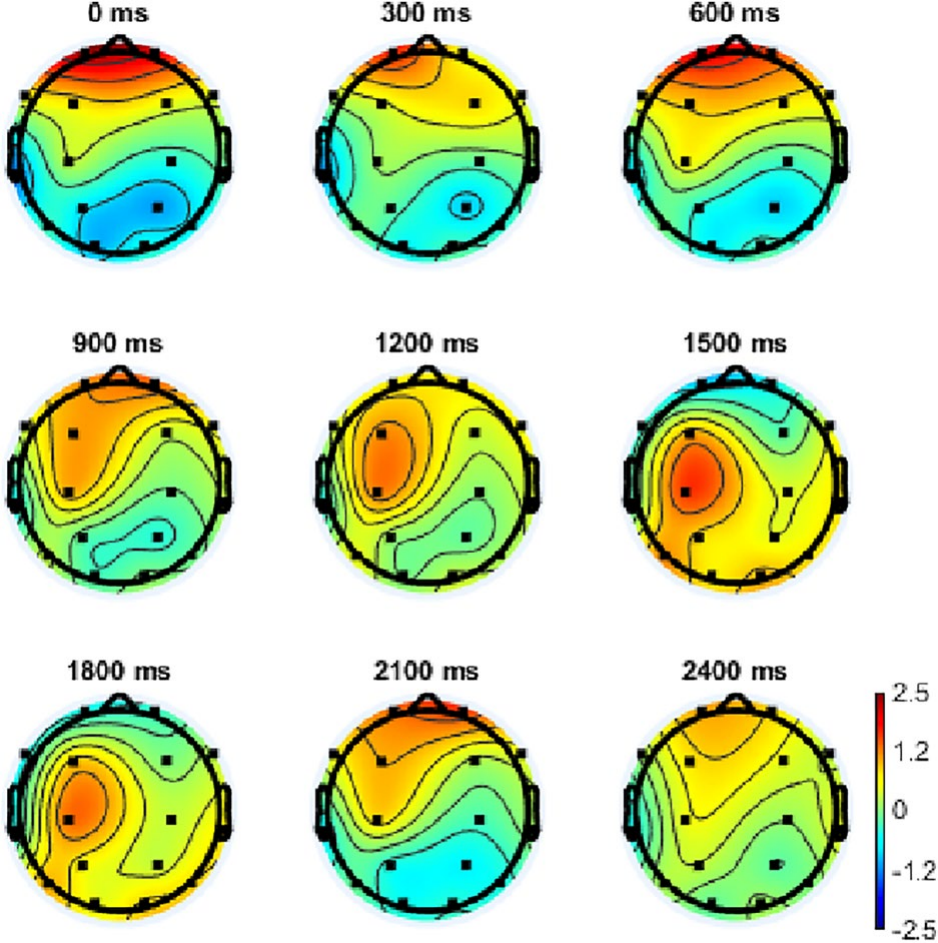
The brain states series of MEng design engineering students.

The results of MEng design engineering students (Fig. 7) revealed that Fp1 and Fp2 were active initially and reduced slightly in the following series. Then, between 900 and 1200 ms, F3 and C3 were active. In 1500 ms, C3 was active strongly. Between 1200 and 1800 ms, the whole brain is active and F3 and C3 were the most active brain areas during this period.

## Discussion

### ERPs results

ERPs are small voltages generated in the brain, which can quantitatively reflect the brain’s response to a specific cognitive event or stimuli. The highest EPR result of MFA visual art students is 1710 ms while the highest EPR result of MEng design engineering students is 1451 ms. This means MEng design engineering students may need 1451 ms to respond to creative tasks while MFA visual art students may need 1710 ms to respond to creative tasks. Because MEng designengineering students need less time to react to creative processes than MFA visual art students, we summized that MEng design engineering students can respond to creative processes faster than MFA visual art students. In the study, MFA visual art students were recruited to represent the artistic-mindset students while MEng design engineering students represent the engineering-mindset students. Therefore, this result on the highest ERP indicated that the speed of artistic-mindset students onresponse to creative tasks was slower than the engineering-mindset students.This may be because although both artistic- and engineering-mindset students need to develop creative ability, in their education, engineering-mindset students were trained more on product creativity while artistic-mindset students were more familiar with personal creativity^40^. The ERPs results of this study were different from the results of Ahad et al.^32^ who supported the view that the highest ERP of engineering-mindset students is in N400. The difference between the Ahad et al.^32^ and this study may be that this study selected a longer range of ERP periods.

It is notable that this finding is not supported by direct behavioral analysis, such as ideation behavior assessment or creativity scoring. For the ideation behavior assessment, our findings indicate that MEng design engineering students have a faster reaction speed in their brains. This is a result from cognitive activation levels instead of the observable behavior level. There is still some period needed between the reaction in the brain and the reaction in observable behavior. During that period, more factors may need to be included such as external environment, which makes the speed of reaction on observable behavior and that of cognitive activation not consistent. Thus, the ideation behavior (observable behavior) is not what we focused on. For the creativity scoring, our findings relate to MEng design engineering students having a faster reaction speed in cognitive activation in a creative process. We do not focus on if this faster reaction speed will lead to a higher creativity scoring. Thus, the creativity scoring is also not what we focused on. However, these are potential research directions in a future study. To be specific, in the future, we can collect more direct behavioral data related to participants’ creative processes, such as ideation behavior assessment and creativity scoring, in this way to further analyse what are the different performances between artistic- and engineering-mindset students and provide more explanation on the different cognitive performance between artistic- and engineering-mindset students.

The highest ERP existed in different locations between artistic- and engineering-mindset students. In terms of artistic-mindset students, the highest ERP was mainly located in the Fp1, Fp2, and F3. Also, F7 and O2 were active but not as strong as the former three areas. This indicated that artistic-mindset students relied on the frontal lobe to generate creative ideas. The frontal lobe has been verified to be related to creative processes^40^ through integrating episodic and semantic memory^41^. Episodic and semantic memories are two categories of long-term memories (LTM). Therefore, the results indicated the contribution of LTM to artistic-mindset students in creative processes.

In terms of engineering-mindset students, the highest ERP was mainly located in the C3, P3, T6, O1, and O2. This indicated that engineering-mindset students relied on parietal, temporal, and occipital lobes of brains to finish their creative processes. This result partly supported the existing research where the researchers have supported that the O1 and O2 were active in a creative process of engineering-mindset students^42^. However, this study included more active brain areas, such as parietal and occipital lobes. The parietal and occipital lobes were mainly related to the creative process by increasing the originality of the ideas^42,43^. This indicated that in a creative process, engineering-mindset students were more likely to focus on the originality of the ideas which is one criterion of creativity.

The different ERP results supported the effect of different mindsets on creative processes^8^. Comparing the active areas of the brain between artistic- and engineering-mindset students, more areas were coupled in engineering-mindset students’ brain areas (parietal, temporal, and occipital lobes) compared with that of artistic-mindset students (the frontal lobe). The coupling can increase the possibility of creativity^42,44^ which indicated that the engineering-mindset students may have more possibility to generate creative ideas. This is different from the results of artistic-mindset students whose active brain area was mainly the frontal lobe.

### Power spectral density (PSD)

In this study, Theta wave band was represented by 6 Hz; Alpha wave band was represented by 10 Hz; Beta wave was represented by 22 Hz. From the PSD results, it can be found that for artistic-mindset students, Fp1 was the active brain areas of 6 Hz, 10 Hz, and 22 Hz. This is consistent with the results of ERPs that Fp1 was one of the active areas in creative processes.

The PSD result also indicated that Theta, Alpha, and Beta waves were active in the left frontal lobe area during a creative process for artistic-mindset students. This is consistent with the general conclusions that creative processes were related to the left frontal area^45–47^. Also, this result can support the view that the creative process of artistic-mindset students was related to the increase of theta wave in the frontal lobe^46^, alpha wave^31^ in the frontal lobe^42,48^, and beta wave^48^. This study further supports this frontal lobe is the left frontal lobe.

It can also be found that the core active areas of engineering-mindset students during creative processes were Fp1 and Fp2 for all three band waves (Theta, Alpha, and Beta waves). This is consistent with the results of existing research that both artistic- and engineering-mindset students relied on the frontal lobe to generate creative ideas. However, this study further supplied that the engineering-mindset students included both left and right frontal lobes activities while artistic-mindset students mainly included the left frontal area activity.

In addition, in each band wave, engineering-mindset students were identified more active brain areas than that of artistic-mindset students in creative processes. For example, in the Theta wave range of engineering-mindset students, P3, P4, O1, O2, T5, and T6 have shown an activity. This indicated that parietal, temporal, and occipital lobes areas were active in Theta wave of engineering-mindset students. Theta waves of artistic-mindset students reported an activity in the left frontal lobe area. In creative processes, Beta waves of engineering-mindset students suggested an activity in P3, which means the parietal lobe was active, while Beta waves of artistic-mindset students indicated an activity in the left frontal lobe area.

### Brain states series

This study also detected the brain states series in each 300 ms among the first 2400 ms. For the results of artistic-mindset students, it can be found that in creative processes, the F3 was the most active brain area. The F3 kept increasing until 1800 ms and then reduced slightly. This most active time is consistent with the ERP results which was 1710 ms. This further supports the results from PSD where the frontal lobe was related to the creative process. The O2 was also active but the activation level was lower than the F3. This indicated that for artistic-mindset students, although creative processes were mainly related to the frontal lobe, the occipital lobe area also assisted the creative processes of artistic-mindset students by providing visual help. This is also consistent with the characteristics of artistic-mindset students whose education was focused on visual delivery^49^.

In terms of engineering-mindset students, it can be found that the active brain areas were more than that of artistic-mindset students. This result is consistent with the ERPs and the PSD results. At 0ms and 600 ms, the Fp1 and Fp2 were active. In the 1500 ms, the C3 was active. This is consistent with the ERP results of the study which indicated the highest ERPs is in 1451 ms.

Based on the results, it can be found that the active levels of engineering-mindset students were lower than that of artistic-mindset students, while the active brain areas of engineering-mindset students in creative processes were more than that of the artistic-mindset students. This leads to the assumption that the performance of creative processes is more likely to be affected by active brain areas instead of the active levels of specific brain areas.

## Conclusions

The results of this study can fill the following three research gaps. Firstly, prior research mainly relates to detecting which part of the brain or which band wave was more active when artistic- and engineering-mindset students finish a creative task. However, there are still more neuroscience characteristics worth detecting such as ERPs, power spectral density, and brain states series. This study filled this gap in existing research, addressing the different performances between artistic- and engineering-mindset students in creative processes. The study found engineering-mindset students have quick thinking in terms of generating creative ideas as fluency. This provided more insight on idea-generation processes of engineering-mindset students. Thirdly, the study supported the evidence base that the active brain areas of artistic-mindset students in creative processes are fewer than that of the engineering-mindset students. This provides more evidence that the mindset can affect the active levels of the brain areas in a creative process.

The contribution of this study can be divided into theoretical and practical implications. In terms of theoretical implications, firstly, our study can supply the findings of other studies. Firstly, the results of this study compared the ERPs results, power spectral density, and brain states series results between artistic- and engineering-mindset students with the help of EEG. This addresses a gap in existing research where ERPs results, power spectral density, and brain states series results from EEG have not been fully explored. In addition, our study can be linked with other studies. For example, this study provides a cue to other researchers. When they applied EEG to understand creative processes or mindsets, brain waves and active brain areas were not the only brain activities that can be focused on. Additional areas of brain activity such as ERPs, PSD, and brain states are also worth detecting.

In practical aspects, this study can contribute to design practitioners and design educators. This study can help educators to better understand the differences between artistic- and engineering-mindset students when they are engaged in creative processes. This understanding can trigger more suitable education strategies to stimulate students’ creative abilities. For example, the study has found that when engineering-mindset students try to generate creative ideas, they are more likely to focus on the originality of the ideas which is one criterion of creativity. However, creativity also includes more criteria such as value. Therefore, during teaching, educators can consciously introduce more strategies for thinking about the value of design, in this way to help engineering-mindset students consider how to make their creative ideas valuable.

The study revealed the cognitive activations of artistic- and engineering-mindset students. This provides a way for educators to integrate different thinking methods based on different mindsets students. For example, the results of this study supported thatthe education of artistic-mindset students was focused on visual delivery, which led to the activity of frontal and occipital lobe areas in artistic-mindset students’ creative processes. The results of this study also supported thatthe education of engineering-mindset students made them more likely to focus on originality in creative processes. The two findings bring the potential for interdisciplinary learning. Educators can introduce the focus point tendency during the creative processes to artistic- and engineering-mindset students, to help them understand which kind of things they are more likely to focus on during the creative processes. When artistic- and engineering-mindset students cooperate with each other, they thus can know their potential trends in thinking and learn from each other.

## Limitation and future research

During the research process, some limitations existed and reduced the reliability of the study. Firstly, considering the broader areas of art and engineering, the study elected MFA visual arts and MEng design engineering students to represent artistic- and engineering-mindset respectively. Although we tried to validate this representation by asking participants self define whether they think they have an artistic- and engineering-mindset, this classificationis not validated through established tests. This may lead the reported cognitive distinctions merely reflect group-specific characteristics rather than broader mindset-related cognitive functions and reduce the reliability of the results. Therefore, in the future, whether this representation is effective needs to be further verified. Secondly, the study only recruited fifteen participants for each kind of mindset. This decision was made based on the existing research. Also, to mitigate the risk on small sample size, we tried to ask participants to finish more trials of the tasks, in this way to increase the data samples. However, the participants number may be statistically not enough and robust to generate reliable findings. In the future, more participants from various backgrounds, ages, and cultures will be recruited to increase the reliability of the study. Thirdly, because the study focused on understanding the creative processes of artistic- and engineering-mindset students with the help of EEG, the study did not calculate the creative scores of the generated creative ideas from artistic- and engineering-mindset students. In the future, the creative scores of each group of students can be collected and calculated to analysis the creative outcomes performance of artistic-and engineering-mindset students and combined it with the EEG results. Finally, although we tried to control the confounding variables by asking both groups participants only focused on the creative tasks, the confounding variables may still exist, such as such as participants’ age, state of consciousness, physical and mental activity and the presence of different biological, environmental stimuli and pharmacological agents. This mayundermine the robustness and generalizability of the findings. In the future, we may need to control some more factors to reduce the confounding variables.

## Methods

### Participants

To achieve the research aim, fifteen Master of Fine Arts (MFA) visual arts (7 males, 8 females, aged from 22 to 25) and fifteen Master of Engineering (MEng) design engineering students (8 males, 7 females, aged from 22 to 25) were recruited. The decision on the enrollment of fifteen MEng and fifteen MFA students, thirty participants in total, was in line withexisting research, such as Fink et al.^50^, Benedek et al.^51^, Yang et al.^52^, and Van Eekeren et al.^53^. These studies all use thirty participants in total to understand the creative processes of designers through neuroscience methods.

All thirty participants have normal or corrected-to-normal vision and do not have diagnosed psychiatric discords, color blindness, or other barriers in reading from computers and wearing EEG devices. All participants did not take in caffeine, unprescribed medicine, or alcohol in the previous three days before taking part in this study. An information sheet was available to participants before the study, and opportunities were provided for participants to ask any questions for clarification. All participants had signed the consent forms providing permission to use their study data.

Master students were selected as the participants, as they have a higher-education background and their mindsets are more representative compared with undergraduate students. This can reduce the bias generated from the professional levels of knowledge. In addition, both arts and science are large areas and include various majors^7,10^. This study selected students from MFA visual arts to represent the artistic-mindset students. This is considering that (1) under the education of MFA, students are more likely to form an artistic mindset^54^; (2) visual arts students focus on the virtual delivery, follow some design processes, and have the requirement on creativity^49^. MEng design engineering students were selected to represent the engineering-mindset students, considering (1) under the education of MEng, students are more likely to form an engineering mindset^55^; (2) design engineering is a major which needs support from creativity to achieve innovation in problem-solving processes^56,57^. All students have been introduced to the artistic and engineering mindsets before the task. Artistic mindsets are expressed as an open-minded and creative mindset to think about and express the world and life through perspectives of aesthetics; Engineering mindsets are explained as a problem-solving mind, where people try to understand how things work and solve problems in a creative but reasonable way. All the fifteen MFA students self-reported their mindsets as artistic mindsets, and all the fifteen MEng students self-reported their mindsets as engineering mindsets.

## Methods

### Creative tasks

This study employed the alternative uses tasks (AUT) as the creative tasks^58^. AUT has been applied to reflect creative processes by various researchers^59–61^. In AUT, participants were asked to think of a use of the given everyday objective that only a few people would think of (for example, ball—nose of a clown). Each participant needs to finish thirty trials of AUT. Fifteen trials were presented with a form of everyday objective in words and fifteen trials were presented with a form of everyday objective in images.

The reason why both words and images were selected is to reduce the EEG characteristics bias generated from images and text recognition processes. Each image (or word) was presented once in a random order. The words and graphics were collected from Stevens Jr and Zabelina’s^62^ study. The corresponding graphics were collected using the BaiduImage search engine (https://image.baidu.com/), a common image search engine in China. All the images were resized to 500 × 500 pixels.

### Neuroscience technology selection

This study aimed to compare the creative process of artistic- and engineering-mindset students through neuroscience technologies. The following criteria were used to select the suitable neuroscience technologies: (1) the technology should be a non-invasive technology to protect the safety of participants. (2) The technology needs to have a good temporal resolution to collect dynamical brain status changes. Based on the two criteria, EEG was selected as the target neuroscience technology.

It is notable that EEG has a low spatial resolution (5–9 cm voxels). This indicated that EEG may not report where the task-related neurons are active accurately^63^. However, EEG has been used to understand the active brain areas in creative processes in various research^42,64,65^, which can support the feasibility of EEG in understanding the active brain areas.

#### Devices

The Neurofax EEG-9200 system, a medical-grade EEG device, was used to record the EEG signals (NIHON KOHDEN, Tokyo, Japan). The Neurofax EEG-9200 system includes 16 scalp and 2 mastoid Ag/AgCl electrodes (M1, M2) mounted according to the 10/20 system. Channels Fp1/Fp2/F7/F8/F3/F4 report signals on the frontal lobe, C3/C4/P3/P4 report signals on the parietal lobe, T3/T4/T5/T6 report signals on the temporal lobe, and O1/O2 report signals on the occipital lobe. Considering that the study is also interested in potential hemispheric differences, midline electrodes such as FZ, CZ, and PZ, were not included^34^. Neurofax EEG-9200 system also includes an EEG measurement system, an amplifier, and EEG results viewing software – NT9200 Digital electroencephalograph. The impedances of all EEG channels were below 5 kΩ. The data were sampled at 1000 Hz. The EEG tasks were generated and presented with the help of E-Prime 3.0. All tasks were presented on a computer screen (35.89 × 24.71 cm with a resolution of 2560 × 1600). The data were collected and stored in the NT9200 Digital electroencephalograph software.

#### Protocol

Participants were first asked to fill out a 5-min questionnaire to collect their basic information such as age, gender, and whether they had taken medicines during the last three days. Then, participants wore the EEG device with the help of researchers. After that, participants were asked to finish thirty AUT trials. The order of the thirty trials was at random. Each trial started with a fixation period, when a light-grey black fixation cross was presented in the middle of the screen jittering between 2 and 5 s. After that, the word or image was displayed and remained in the middle of the screen for up to 5 s. During this period, participants were asked to recognize the word or image in 5 s without verbalisation. To prevent participants from generating ideas in the recognition stage, the recognition interface presented a green font “Recognition” text at the top of the image or text. If participants finished recognition before timeout, they could hit the space key on the keyboard to the next interface(idea generation interface). If the 5 s ran out, the interface would jump to the next interface (idea generation interface)automatically. When the interface jumps to the idea generation interface, an event mark (Mark 1) is taken automatically in E-prime 3.0.In the next interface, participants had up to 20 s to “think of a use of the given object that only few people would think of but not verbalise”. If they found a solution before the timeout, they could hit the space key on the keyboard to the next interface. If the 20 s ran out, the interface would jump to the next interface automatically. When the interface jumps to the next interface, an event mark (Mark 2) is taken automatically in E-prime 3.0. The whole study took about 30 min to complete. The whole protocol is displayed shown in Fig. 1.

Informed consent was obtained from all subjects. All experimental protocols were approved by the Science, Engineering and Technology Research Ethics Committee (SETREC) of Imperial College London. Also, all experiments were performed in accordance with the Declaration of Helsinki.

### Data analysis

This study focused on the EEG results. Event-related potentials (ERPs), power spectral density (PSD), and brain state series analysis were conducted. ERPs are small voltages generated in the brain, which can quantitatively reflect the brain’s response to a specific cognitive event or stimuli. ERPs analysis has been commonly used in analysing EEG data and has been verified by various researchers.ERPs in this study can help researchers to understand how much time that artistic and engineering mindsets students need to first respond to the creative tasks. This may further explain why artistic and engineering mindsets students need different time in creative processes.The power spectral density (PSD) analysis is a method to analyse signals’ power content through frequency. Various spectra range of waves (such as alpha and beta) has been verified to relate to different mind activities. The PSD thus can be used to explain neural mechanisms of artistic and engineering mindsets in creative processes. Brain state series were used to understand the dynamical brain-status changes, which can help researchers further understand the dynamic changes of brain activities in depth.

The MATLAB R2022b and EEGLAB plugin were used to analyse the data. The EEG data of fifteen MEng students (or fifteen MFA students) data were imported firstly. In the study, Mark 1 was taken when the interface jumps to the idea-generation interface while Mark 2 was taken when the idea-generation interface jumps to the next interface. These marks allow researchers to identify the idea-generation processes (the period between Mark 1 and Mark 2) of participants in each trial. Therefore, researchers can extract all idea generation processes EEG data as epochs in EEGLAB.

Using the “Automated artifact rejection function” in EEGLAB, which is based on the “Clean raw data” EEGLAB plugin, bad data channels and bad portions of data were marked automatically^33^. EEG signals marked as “artifacts” were removed from analysis. A 50 Hz notch filter was applied to negate the interference of the electrical mains. Following that, the signals were passed through a band-pass filter with a pass-band of 0.1–100 Hz^34,35^. The reference electrodes were placed on the left and right mastoid (M1 and M2).

Three analyses were conducted including ERPs analysis, power spectral density, and brain states series. The ERPs analysis was conducted based on the “With scalp maps” function in EEGLAB, which can plot the average ERP of all dataset epochsand display the latency of maximum ERP data variance. The latency of maximum ERP data variance between fifteen MEng students and fifteen MFA students was compared. The power spectral density analysis was achieved via the “Channel spectra and maps” function in EEGLAB, which can plot the channel spectra and associated topographical maps. Considering the spectra range of theta waves was in 4–8 Hz, Alpha waves was in 8–12 Hz, and Beta waves was in 12–30 Hz, the middle spectra number of each band wave was selected to represent each band wave^38^. The power spectral density results on Alpha, Beta, and Theta waves between fifteen MEng students and fifteen MFA students were compared respectively. Brain states series is reported from “ERP map series” function in EEGLAB, which can represent potential distributions at a selected series of times during the epoch. In this study, the results showed the brain states series of each 300 ms in the first 2400 ms. The ERP map series results between fifteen MEng students and fifteen MFA students were compared.

## Data Availability

Correspondence and requests for materials should be addressed to the corresponding author.
